# Draft Genome Sequences of *Rhodococcus* sp. Strains YH1 and T7, Isolated from Explosive-Contaminated Environments

**DOI:** 10.1128/MRA.00097-20

**Published:** 2020-05-28

**Authors:** Paula Istvan, Zeev Ronen

**Affiliations:** a Department of Environmental Hydrology & Microbiology, Zuckerberg Institute for Water Research, Jacob Blaustein Institutes for Desert Research, Ben-Gurion University of the Negev, Sede Boqer Campus, Beer Sheva, Israel; University of Maryland School of Medicine

## Abstract

We report the draft genome sequences for *Rhodococcus* sp. strains YH1 and T7. These strains are both capable of degrading hexahydro-1,3,5-trinitro-1,3,5-triazine (RDX) and were isolated from explosive-contaminated soil and groundwater, respectively. Further genomic analysis might facilitate an understanding of the degradation of RDX and will contribute to the development of bioremediation methods for polluted soil and groundwater.

## ANNOUNCEMENT

The production, use, storage, and dismantling of explosives have resulted in large-scale contamination worldwide. Hexahydro-1,3,5-trinitro-1,3,5-triazine (RDX) is one of the explosives used on a large scale (1). RDX has structures and properties that do not exist in nature, and it is not highly absorbed by soil particles, which results in its leaching and the formation of plumes of groundwater contamination (1). Sources of explosive contamination include production facilities and packing and handling sites, as well as training ranges. In training ranges, unexploded ordnance serves as a long-term source of soil and water pollution (2). Biological treatment through microbial biodegradation is a possible cost-effective treatment for such pollution. However, the presence of many explosives and other cocontaminants like ammonium, nitrate, and perchlorate can hinder this approach (3).

*Rhodococcus* sp. strain YH1 was isolated from a sequencing batch reactor used to simulate the treatment of munition wastewater (4). *Rhodococcus* sp. strain T7 was isolated from RDX-polluted groundwater using passive sampling and enrichment (2). These strains are both capable of degrading RDX, and they were cultivated with RDX as the sole nitrogen source (2, 4). The 16S rRNA gene sequences (GenBank accession no. AF103733 and FJ790675) were both 100% identical to *Rhodococcus* sp. sequences in GenBank. The *xplA* gene was also sequenced to confirm the degradation mechanisms (2, 3). After 48 h of growth of the strains on Trypticase soy agar (30°C), genomic DNA was extracted using the DNeasy blood and tissue kit (Qiagen, Hilden, Germany). The DNA was quantified using a Qubit 3.0 fluorometer (Life Technologies, Carlsbad, CA). The genomic DNA library was prepared using the Nextera Flex DNA library preparation kit (Illumina, San Diego, CA). Sequencing was performed using an Illumina MiSeq instrument to generate 250-nucleotide (nt) paired-end reads. We generated 2,384,946 reads for strain YH1 and 2,787,630 reads for strain T7. The read quality was assessed with FastQC version 1.0.0 (Illumina BaseSpace Labs). The reads were assembled using SPAdes version 3.9.0 (5). Coding sequences (CDSs), mRNAs, rRNAs, tRNAs, genes, and pseudogenes were determined using Prokka version 1.12 (6) and the UniProt database (7). Default parameters were used for all software unless otherwise noted.

The draft genome of *Rhodococcus* sp. strain YH1 is 6,422,517 bp, and that of *Rhodococcus* sp. strain T7 is 9,358,508 bp, distributed in 292 and 389 scaffolds, respectively. The sequence depth coverages for YH1 and T7 are 35× and 52×, respectively. The *N*_50_ values for YH1 and T7 are 40,661 bp and 66,978 bp, respectively. The GC contents for YH1 and T7 are 59.15% and 56.28%, respectively. The annotation of the *Rhodococcus* sp. strain YH1 genome identified 6,047 genes, 5,980 coding sequences, 1 transfer-messenger RNA, 66 transfer RNAs, and 1 clustered regularly interspaced short palindromic repeat (CRISPR). The annotation of the *Rhodococcus* sp. strain T7 genome identified 8,850 genes, 8,785 coding sequences, 1 transfer-messenger RNA, and 64 transfer RNAs.

These genome sequences will improve our knowledge of RDX degradation genes and metabolic pathways. Also, this research may contribute to the development of methods for the bioremediation of polluted soil and groundwater.

### Data availability.

This whole-genome shotgun project has been deposited in DDBJ/ENA/GenBank under accession no. WVTE00000000 for *Rhodococcus* sp. strain YH1 and WXYH00000000 for *Rhodococcus* sp. strain T7. The raw reads are available in the Sequence Read Archive (SRA) under accession no. SRR10816273 and SRR10822287, respectively. The strains' 16S rRNA gene sequences are available under accession no. AF103733 and FJ790675, respectively. The versions described in this paper are the first versions.
